# Validity and reliability of My Jump 2^®^ app to measure the vertical jump on elite women beach volleyball players

**DOI:** 10.7717/peerj.17387

**Published:** 2024-05-17

**Authors:** Alexandre Igor Araripe Medeiros, Geovani Messias da Silva, Francisco Oliveira Neto, Mário Simim, Túlio Banja, Victor S. Coswig, José Afonso, Ana Ramos, Isabel Mesquita

**Affiliations:** 1 Master Program in Physiotherapy and Functioning, Federal University of Ceara, Fortaleza, Ceara, Brazil; 2 Research Group in Biodynamic Human Movement, Institute of Physical Education and Sports, Federal University of Ceara, Fortaleza, Ceara, Brazil; 3 Centre for Research, Education, Innovation, and Intervention in Sport (CIFI2D), Faculty of Sport of the University of Porto, Porto, Portugal; 4 Associated Graduate Program in Physical Education, Federal University of Paraiba, João Pessoa, Paraíba, Brazil; 5 Praia Clube, Uberlândia, Minas Gerais, Brazil

**Keywords:** Biomechanics, Force platform, Microtechnology, Mobile device, Motion analysis, Physical performance, Testing

## Abstract

**Purpose:**

The aim of this study was to assess the reliability and validity of the My Jump 2^®^ app in measuring jump height, flight time, and peak power among elite women beach volleyball players on sand surfaces.

**Methods:**

Eleven elite female beach volleyball players (aged 23.6 ± 6.2 years; weight 66.3 ± 5.8 kg; height 174.4 ± 5.8 cm; with 8.4 ± 4.8 years of professional experience) participated in this study. Each player performed six countermovement jumps in a wooden box filled with sand on a force platform while simultaneously recording a video for subsequent analysis using the My Jump 2^®^ app.

**Results:**

We found excellent agreement for flight time, jump height and peak power between observers (ICC = 0.92, 0.91 and 0.97, respectively). No significant differences between force platform and My Jump 2^®^ app were detected in the values obtained for the three variables (*P* > 0.05). For the force platform and the My Jump 2^®^ app, we found a good agreement measuring jump height and flight time (ICC = 0.85 and 0.85, respectively). However, we only found a moderate agreement for peak power (ICC = 0.64). The difference in jump height showed a limit of agreement between −4.10 and 4.74 cm in Bland-Altman, indicating a high level of agreement between the two measurement tools.

**Conclusion:**

Based on our findings, the My Jump 2^®^ app reveals a valid tool for measuring jump height and flight time of CMJ on sand surfaces. However, more caution is needed when measuring peak power.

## Introduction

Beach volleyball is a team sport characterized by its intermittent nature, fluctuating randomly from short periods of maximal or sub-maximal activity to long periods of moderate and low-intensity activity (Arruda & Hespanhol, 2008; Magalhães et al., 2011; Medeiros et al., 2014). This alternating activity nature arise in game actions as jumps, constituting one of the most determining physical-technical elements in beach volleyball performance (Andrade et al., 2020; Medeiros et al., 2014, 2023; Oliveira et al., 2018). Accordingly, literature within beach volleyball scope has pointed out that jump measures can help coaches monitor training load and understand the activity profile of players (Andrade et al., 2020; Medeiros et al., 2023; Oliveira et al., 2018).

Specifically, the countermovement jump (CMJ) has been one of the most popular tests for assessing and monitoring neuromuscular status in volleyball and beach volleyball (Andrade et al., 2020; Batista, RFd & Guerra, 2008; Oliveira et al., 2018; Sheppard et al., 2011; Sheppard & Newton, 2012).

However, most of the investigations conducted so far have evaluated jump height of CMJ on a rigid surface, even in the case of beach volleyball (Andrade et al., 2020; Oliveira et al., 2018). This could bring specificity issues (*i.e*., ecological validity; Davids, 1988) since the assessment of CMJ on a rigid surface could modify jumping performance (Giatsis et al., 2004; Giatsis, Panoutsakopoulos & Kollias, 2018). For example, beach volleyball players demonstrate performing lower vertical jumping heights when jumping on a sand surface compared to a rigid one (Bisciotti, Ruby & Jaquemod, 2001; Bishop, 2003; Giatsis et al., 2004). However, the kinetics of female beach volleyball players when examined across vertical jump tests executed on sand show a different trend compared to male players (Giatsis et al., 2023).

The compliance of the sand imposes a constraint for an efficient coordinated extension of the lower limb joints and requires higher expenditure of energy to fulfill the demand of maintaining postural stability (Muramatsu et al., 2006; Smith, 2006). As result, reduced application of force and less power production during the propulsion of jumping from a sand surface (Bisciotti, Ruby & Jaquemod, 2001; Bishop, 2003; Giatsis et al., 2004; Impellizzeri et al., 2008).

The assessment of vertical jump height using a force platform (usually *via* take-off velocity or flight time) might be considered as the gold standard (Garcia-Lopez et al., 2013; Glatthorn et al., 2011; Requena et al., 2012). Although this equipment offers higher levels of accuracy and reliability for CMJ measurement, the device depicts many limitations for measuring in field and real-time conditions. Also, issues related to the portability of force platform, their associated costs, and the technical demands to manage the equipment seem to become it unsuitable for most field-based testing.

In this context, applications (apps) for mobile devices have been suggested as alternative tools to measure vertical jumps (Balsalobre-Fernández, Glaister & Lockey, 2015). Among those, the My Jump2^**®**^ app was designed to calculate the jump height from the flight time, which is based on the take-off and landing frames selected by the user (Balsalobre-Fernández, Glaister & Lockey, 2015; Brooks, Benson & Bruce, 2018; Yingling et al., 2018). Both the accuracy and reliability of this app have been tested in different populations, including athletes (Barbalho et al., 2021; Gallardo-Fuentes et al., 2016; Soler-Lopez et al., 2022), elderly (Cruvinel-Cabral et al., 2018a), cerebral palsy soccer players (Coswig et al., 2019), and children (Bogataj et al., 2020). Also, different types of jumps and variables were investigated, including squat jumps, CMJ, drop jumps, flight time, jump height, and asymmetry index (Haynes et al., 2019). Although the My Jump2^**®**^ app consistently presented excellent values of reliability and validity when compared to force platforms and jump mats (*e.g*., Cruvinel-Cabral et al., 2018b; Jimenez-Olmedo et al., 2022; Rago et al., 2018; Soler-Lopez et al., 2022), to date it was not found any scientific investigation that had tested the validity and reliability of the My Jump2^**®**^ app to measuring jump height in sand surface. Furthermore, validity and reliability studies had focus in investigate males (Carlos et al., 2015; Thomas et al., 2019). One study showed that the My Jump2^®^ app had good intra- and inter-session reliability for male and females. The agreement for them were almost perfect (Gallardo-Fuentes et al., 2016). This tool empowers various sports practitioners, including coaches, performance analysts, and teachers, by enabling them to predict injury risks, facilitate talent identification, and seamlessly assess, monitor, and adapt their practices. Moreover, of the My Jump2^**®**^ app reveals a reliable tool, it will enable the inclusion of CMJ assessments in sand surface throughout the practice.

Considering the foregoing, the aim of this study was to assess the reliability and validity of the My Jump 2^®^ app in measuring jump height, flight time, and peak power among elite women beach volleyball players on sand surfaces.

## Materials and Methods

### Participants

Sample size was calculated based on previous investigations dedicated to test the reliability and validity of different tools in expert female players (Gallardo-Fuentes et al., 2016), as well as using the G*Power software (Germany, version 3.1.9.7). Establishing an expected reliability of 0.99, a minimum reliability of 0.7, and an alpha level of 0.05, nine players would be desirable to achieve an estimated power of 0.95. Eleven (*n* = 11) elite women beach volleyball players (23.6 ± 6.2 years; 66.3 ± 5.8 Kg; 174.4 ± 5.8 cm; 8.4 ± 4.8 years of professional experience) participated in this study. The selected players represented the elite of Brazilian beach volleyball, since it includes Olympic players 2021, World Champions 2021, Brazilian Champions, Summer Youth Olympic Games Champions, and under-21 World Champions. The study followed the guidelines stated in the Declaration of Helsinki and was approved by the Institutional Research Ethics Committee of the first author’s institution (Federal University of Ceara) with the following number: 5.336.417. Players were informed about the research scope, as well as the possibility to withdraw from the investigation at any time. Guarantees of confidentiality and anonymity were also explained. Afterwards, consents forms were signed by the participants.

### Design

**Figure 1:**
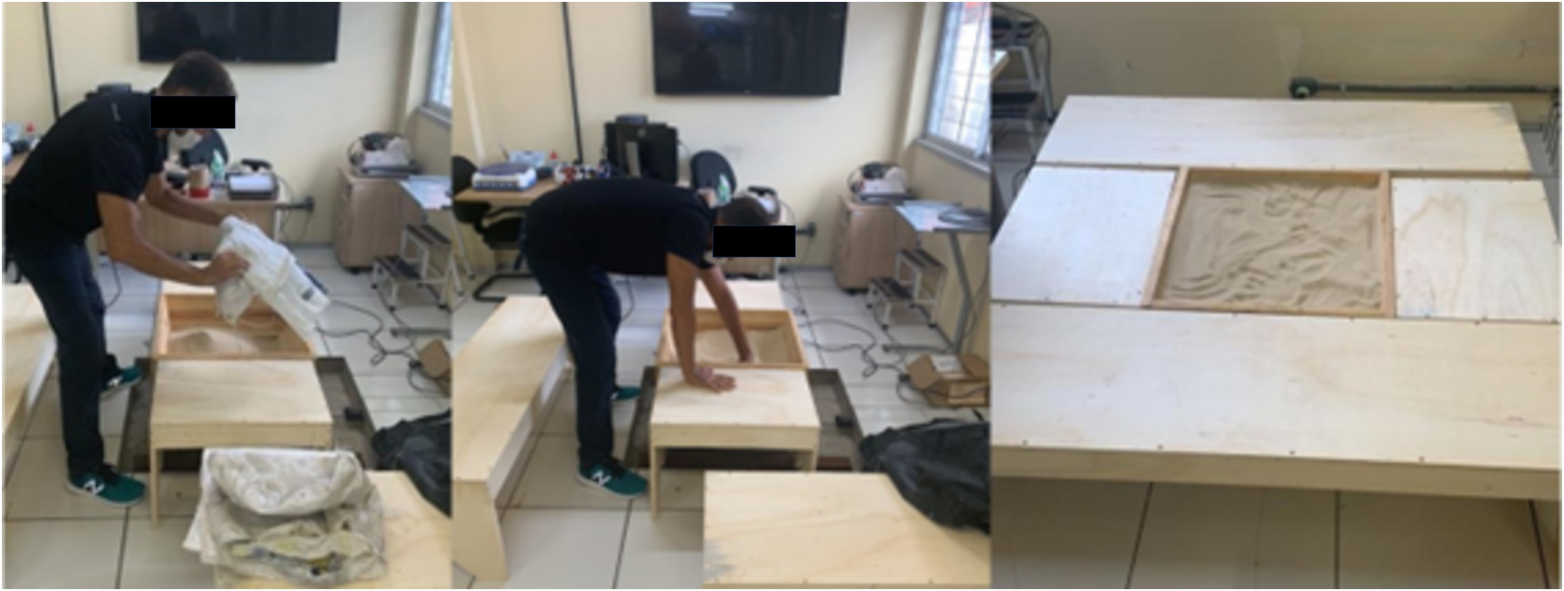
Wooden box filled with sand with wooden structure (50 cm width).

This study followed an experimental design that compared the values of six CMJ (Claudino et al., 2016) for each player between the My Jump 2^**®**^ app and the gold standard force platform (Bertec-Model FP4060-08-1000, COLUMBUS, OH, USA, sampling frequency of 1,000 Hz). All CMJs were performed without arm swing, and inside a wooden box filled with sand (Fig. 1). Globally, 66 CMJ were accomplished, and all participants were familiarized with CMJ technique before testing. Prior the test, the participants completed a standard 20 min warm-up composed of stationary cycling (Monark 817E bicycle at a constant speed of 5.5 m/s^−1^ with 0 W load), lower body dynamic stretches, and vertical jumps (Giatsis, Panoutsakopoulos & Kollias, 2018).

### Countermovement jumps

The participants executed the jump by flexing their knees until reach a comfortable (*i.e*., preferred starting push-off position) (Castagna et al., 2013). To limit possible variations in posture during the jump that could affect the final assessment, the hands had to remain attached to the hip throughout the jump (Aragón, 2000). Subsequently, they were instructed to jump as high as possible, performing a countermovement with their legs straight during the flight phase of the jump (Haekkinen & Komi, 1985). The landing was performed simultaneously with either feet keeping ankle dorsiflexion (Balsalobre-Fernández, Glaister & Lockey, 2015). To avoid some fatigue effect the interval between one attempt and another was 60 s (Claudino et al., 2012).

### Comparison procedures

#### Sandbox force platform

The sandbox was formed by a lower part with perimeter dimensions of 46 × 50 cm, and an upper part with perimeter dimensions of 59 × 63 cm (Giatsis, Panoutsakopoulos & Kollias, 2018). The layer of sand to be used in the sandbox is 40 cm (Fédération Internationale de Volleyball, 2021). The total weight of the box after filling was approximately 120 kg. Before placing the box on the platform, the body mass measurement was performed using a calibrated analogical scale with a precision of 0.01 kg (BALMAK-Model104 A Classe III; Santa Bárbara d’Oeste, São Paulo, Brazil). The scale was placed on a flat and stable surface to prevent any inaccuracies due to tilting or movement. The final weight measurement was recorded after repeated twice to ensure consistency. The average of the two readings was used as the athlete’s body mass in the analysis. Additionally, the force platform was reset with the sandbox on top and then the same body mass value was confirmed after the participant was on top. The same value of body mass was confirmed after placing the box on the platform. Four wooden benches with a width of 50 cm were fitted around the sandbox to ensure the safety of participants in case of any imbalance on landing. The sand was collected on the beaches of Fortaleza in the state of Ceara, Brazil, without any type of organic material in their composition. Particle size distribution was determined (Gee & Bauder, 1986) using chemical (sodium hexametaphosphate) and physical (fast agitation for 10 min) dispersion. The clay fraction was quantified by the pipette method, the sand fraction by sieving, and silt fraction by difference between the total sample of oven-dried fine earth and the sum of sand and clay. We used the sand fractions (0.25–0.1 mm) according to Soil Survey Division Staff (Ditzler, Scheffe & Monger, 2017). The force platform was connected to a computer equipped with the software to analyse the force data (Bertec Digital Acquire 4.0.12.411). The software recorded data at a sampling frequency of 1,000 Hz.

Different strategies were used to evaluate the variables of this study. In relation to evaluation in force platform, the jump height was calculated from the impulse-moment theorem which allows the jumper’s take-off speed calculated by double numerical integration of force (Fernandes et al., 2021). From the takeoff speed it is possible to determine the height of the jump through the equation (V_off_^2^)/2g, (where (V_off_) is takeoff speed and (g) is gravity acceleration. The power was estimated from the product of the participant’s weight and takeoff. Flight time was defined as the time interval during the aerial phase of the jump. The beginning of the jump was defined from the instant that the force values on the platform presented a value equal to zero until the first force value detected when the feet contact to the ground. Time series were analyzed using the DasyLab^®^ software version 11 (National Instruments, Dublin, Ireland) and filtered using a 10 Hz fourth-order Butterworth low-pass filter (Y) implemented in the software. Each CMJ valid was considered as a single case in database.

#### *My Jump 2*^®^ app

The jumps were recorded by the same researcher with a mobile (cell) phone (iPhone 11 promax; Apple, Cupertino, CA, USA) at a sampling rate of 240 Hz, and 1,920 × 1,080 of pixels resolution using the My Jump 2^**®**^ app. The iPhone 11 facing the participant (in the sagittal plane) was positioned on top of a tripod ~ 1,5 m from the force platform at floor level and zoomed in on the feet of the participant. The My Jump 2^**®**^ app was designed to calculate the time (in ms) between two frames selected by the user and subsequently jump height, flight time and peak power (Balsalobre-Fernández, Glaister & Lockey, 2015). The app calculates the Jump height through a simple equation 1/8 gt^2^ where, (g) is the acceleration of gravity and (t) is the time. The flight time is determined by the interval between takeoff and landing that is defined by the user when watching the recorded data. The flight time is estimated based on the acquisition frequency of the cell phone camera (240 Hz) where in our case, each frame corresponds to approximately 0.004 s. Power output is estimated in the app from the product of the participant’s weight (F) and the takeoff speed (V_off_). The takeoff speed is estimated from the flight time (t) and the gravity acceleration (g) using the equation (gt/2) (Balsalobre-Fernández, Glaister & Lockey, 2015).

### Statistical analysis

The normality of data was tested using the Shapiro–Wilk test. Descriptive data are presented as mean and standard deviation (mean ± SD). The reliability of observations with the My Jump 2^**®**^ app was conducted by two observers. Regarding the reliability on force platform, it was assessed through a set of statistics testing the level of agreement and the magnitude of errors (Courel-Ibanez et al., 2019). To measure inter-rater agreement, we calculated the Spearman correlation coefficient (r), the intraclass correlation coefficient with two-way random effects (ICC2,1), and the concordance correlation coefficient (CCC). The coefficient of determination (R^2^) was used to explain how much the variability of one factor was caused by its relationship to another factor. The factors utilized in this study were jump height, flight time, and peak power, which were measured by Observer 1, Observer 2, and the force platform. The following criteria was adopted to interpret the magnitude of the correlation: “trivial” (r < 0.1), “small” (0.1 ≤ r < 0.3), “moderate” (0.3 ≤ r < 0.5), “large” (0.5 ≤ r < 0.7), “very large” (0.7 ≤ r < 0.9), “nearly perfect” (0.9 ≤ r < 1), and “perfect” (r = 1) (Hopkins, 2016). An ICC <0.5 was considered as “very poor”, between 0.5 and 0.75 as “moderate”, between 0.75 and 0.90 as “good”, and >0.90 as “excellent” (Koo & Li, 2016). The interpretation of CCC followed the (Altman, 1990) classification: poor (<0.2) and excellent (>0.8).

The Bland-Altman plot was used to measure the mean difference between force platform and the My Jump 2^**®**^ app (mean of the two observers). The Bland-Altman plot provided the bias between methods and its limit of agreement with a 95% confidence interval (CI).

The absolute and relative reliability of a measure in comparison to its true value was analyzed by calculating the standard error of measurement (SEM) as follows: SEM = SD_diff_/√1−ICC where SD_diff_ was the standard deviation of the mean differences between observers and force platform. SEM results were classified as good (SEM = < 5.0%), moderate (SEM = 5.0–9.9%) or poor (SEM ≥ 10.0%) (Claudino et al., 2021). The coefficient of variation (CV), expressed as a percentage, indicates the level of variability within a set of measurements. To calculate the CV, we used the mean square root. Additionally, we used the adjusted bootstrap percentile (BCa) method to estimate the 95% confidence intervals for the SEM and CV (DiCiccio & Efron, 1996).

The smallest worthwhile change (SWC) was assumed by multiplying the SDdiff by either 0.2 (SWC0.2), indicating the typical small effect or 0.6 (SWC0.6), showing an alternative medium effect (Hopkins, 2000). The ability of the tests to detect a change was rated as “good,” “satisfactory,” or “marginal” when the SEM was below, similar, or higher than the SWC, respectively (Hopkins, 2000). The minimal detectable change (MDC95) of comparisons was determined as MDC95 = 1.96 * SEM * √2 (Weir, 2005). This measure represents the 95% confidence interval of the difference in the score between paired observations (Weir, 2005). This indicator is interpreted as the minimal change required for a given variable so that sufficient confidence for a practically relevant change is provided (Hopkins, 2000).

To determine whether the difference between the My Jump 2^**®**^ app *vs* force platform indices the Mann-Whitney test was used, with confidence intervals (CI 95%). Effect sizes for significant comparisons were calculated using drank biserial correlation (Kerby, 2014). The rank biserial correlation can be calculated using the formula: r = 1 − (2U)/(n1 * n2), where “U” represents the U statistic, and “n1” and “n2” denote the sample sizes of the respective groups alpha level was set at *p* ≤ 0.05. We used Jamovi 2.3.28 (https://www.jamovi.org/download.html) and GraphPad Prism 8 (GraphPad Software, La Jolla, CA, USA) to conduct the analyses.

## Results

Table 1 shows the descriptive statistics of CMJ variables obtained with the My Jump 2^**®**^ app (both observers) and force platform. Values are mean ± standard deviation (SD).

**Table 1:** Descriptive values of countermovement jump (CMJ) kinematic variables obtained with the My Jump 2^®^ app (both observers) and force platform.

Variable	Observer 1 (My Jump 2^®^ app)	Observer 2 (My Jump 2^®^ app)	Force platform
Jump height (cm)	33.99 ± 4.03	33.80 ± 4.27	34.22 ± 4.15
Flight time (ms)	525.52 ± 31.86	523.93 ± 34.07	518.85 ± 31.67
Peak power (W)	1,692.36 ± 367.06	1,682.59 ± 368.70	1,694.52 ± 301.69

**Note:**

Values are mean ± standard deviation (SD).

Table 2 presents the results between-observer reliability to My Jump 2^**®**^ app and, My Jump 2^**®**^ app and the force platform for jump height, flight time and peak power. The differences between observers for My Jump 2^**®**^ in all variables were very small (ES ranging from 0.03 to 0.05).

**Table 2:** Reliability analysis between the My Jump 2^®^ app *vs* force platform.

	Between-observers Reliability—My Jump 2^®^ app			Between-My Jump 2^®^ app and force platform		
	*Jump height (cm)*	*Flight time (ms)*	*Peak power (w)*	*Jump height (cm)*	*Flight time (ms)*	*Peak power (w)*
**U statistic (*p*-value)**	2,117.50 (0.7839)	2,117.50 (0.7960)	2,159.50 (0.9346)	1,865.00 (0.1547)	2,062.50 (0.6006)	2,118.00 (0.7865)
**Paired diff. (CI-95%)**	0.18 [−1.24 to 1.61]	1.59 [−9.77 to 12.95]	9.76 [−116.93 to 136.46]	−0.32 [−1.74 to 1.09]	5.87 [−5.14 to 16.88]	−7.04 [−122.40 to 108.31]
**Paired ES**	0.028	0.028	0.008	−0.0053	0.144	−0.028
**ICC (CI-95%)**	0.92 [0.88–0.94]	0.91 [0.87–0.94]	0.97 [0.96–0.98]	0.85 [0.78–0.90]	0.85 [0.77–0.90]	0.64 [0.50–0.75]
**CCC (CI-95%)**	0.92 [0.87–0.95]	091 [0.86–0.94]	0.97 [0.95–0.98]	0.85 [0.76–0.90]	0.85 [0.76–0.90]	0.63 [0.47–0.76]
**SEM (CI-95%)**	1.18 [0.98–1.38]	9.68 [8.02–11.30]	62.40 [50.8–73.1]	1.60 [1.32–1.86]	11.90 [9.77–13.80]	200 [165.00–233.00]
**CV (%) (CI-95%)**	3.49 [2.87–4.07]	1.84 [1.53–2.15]	3.70 [3.01–4.37]	4.68 [3.85–4.49]	2.27 [1.87–2.63]	11.80 [9.77–13.80]
**MDC (CI-95%)**	3.27 [3.191–3.351]	26.83 [26.09–27.57]	172.96 [172.416–173.512]	4.43 [1.30–7.57]	32.99 [9.66–56.31]	554.37 [162.37–946.37]
**SWC 0.2 (CI-95%)**	2.74 [−0.56 to 6.04]	0.34 [−0.07 to 0.74]	17.67 [−3.65 to 39.02]	0.45 [−0.09 to 1.00]	3.35 [−0.69 to 7.40)	57.06 [−11.77 to 125.90]

The ICC between observers, as well as among the My Jump 2^**®**^ app *vs* force platform, was considered good in relation to jump height and flight time (observers: 0.92 and 0.91, the My Jump 2^**®**^ app *vs* force platform: 0.85 and 0.85). However, in what peak power regards, the ICC was moderate between the My Jump 2^**®**^ app and force platform (0.64).

**Figure 2:**
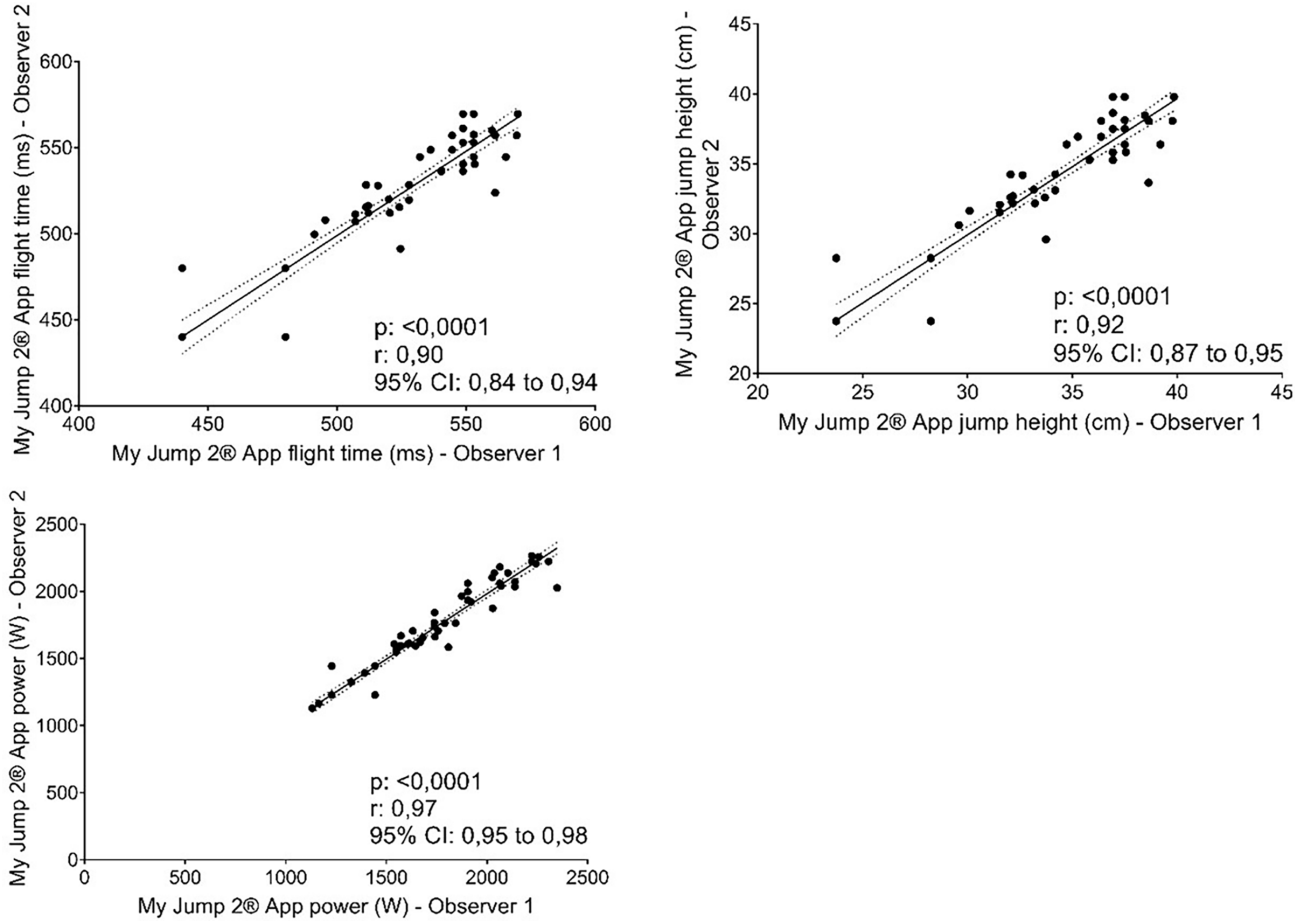
Correlation between two observers using the My Jump 2^®^ app.

Correlations between two observers for the My Jump 2^**®**^ app showed almost perfect relationship (Fig. 2; r = 0.90 to 0.96; R^2^ = 81.00% to 94.00%).

**Figure 3:**
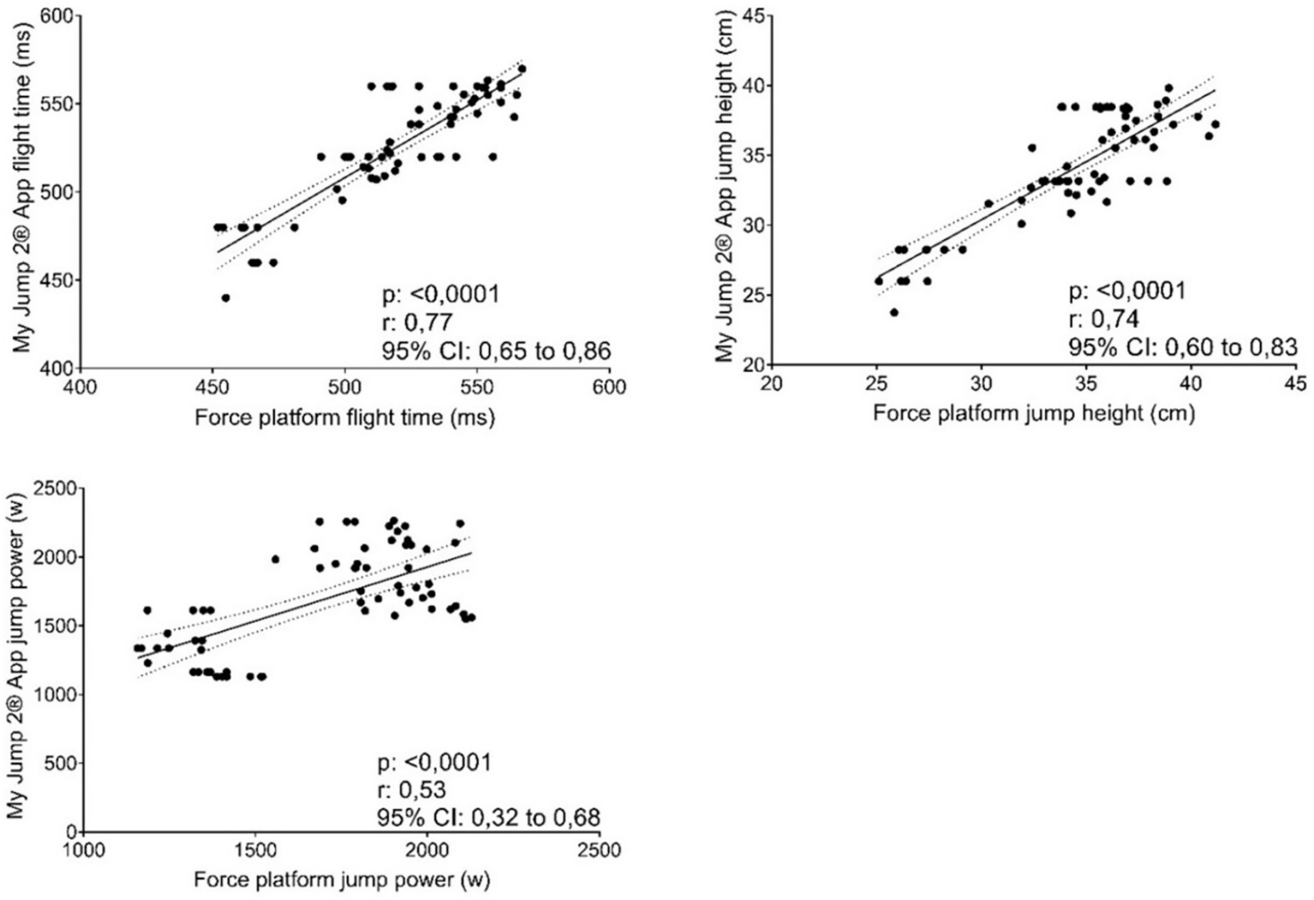
Correlation between two observers mean using the My Jump 2^®^ app and force platform.

The force platform and the My Jump 2^®^ app correlations for jump height, flight time and peak power showed large relationship (Fig. 3; r = 0.52 to 0.77; R2 = 28.00% to 60.00%).

**Figure 4:**
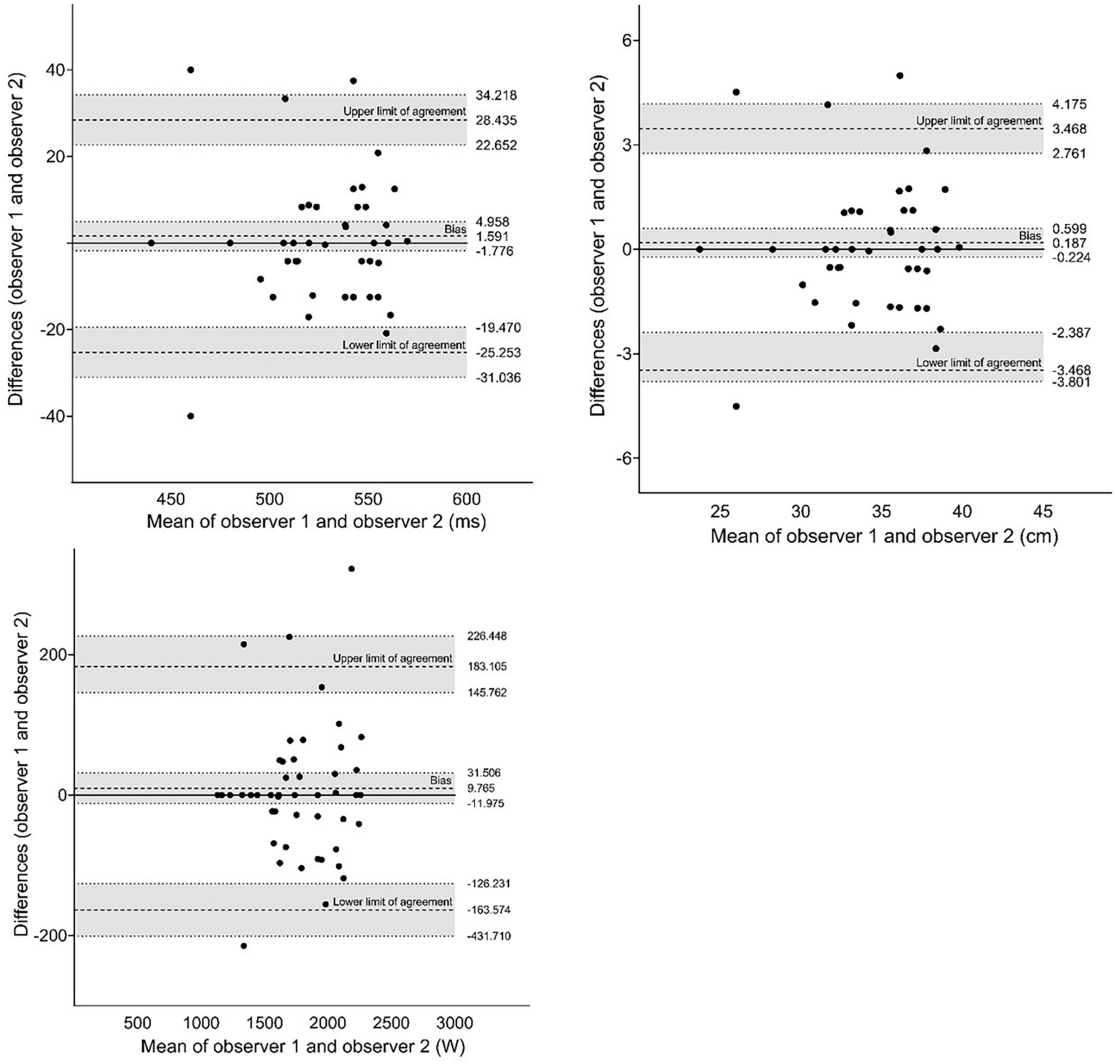
Agreement analysis between two observers for jump variables using the Bland-Altman method.

Figure 4 shows the results of the agreement analysis between the two observers using the My Jump 2^®^ app. We did not observe any systematic bias in the variables. The difference between observers showed acceptable values.

**Figure 5:**
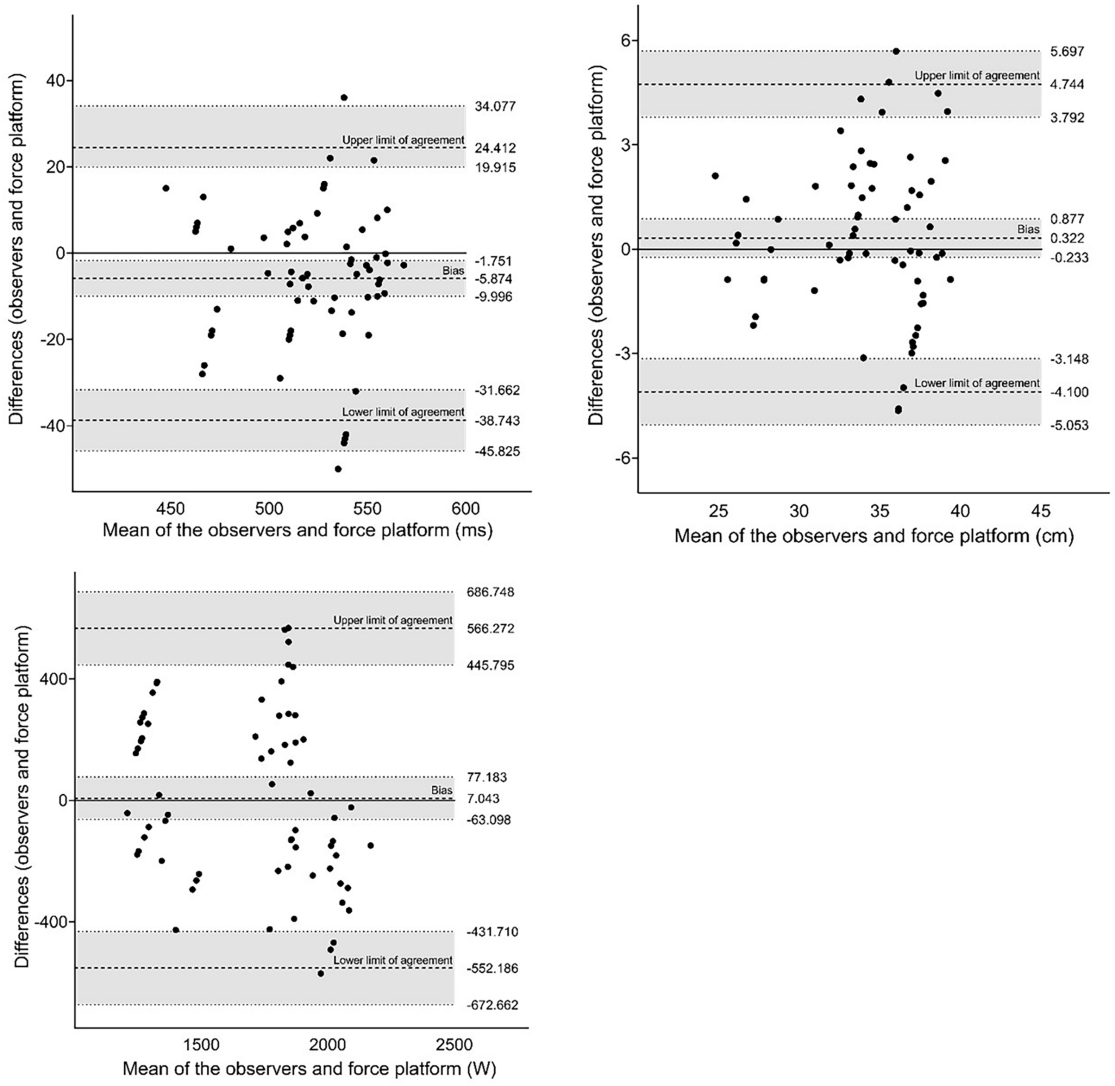
Agreement analysis between the My Jump 2^®^ app and force platform for jump variables using the Bland-Altman method.

The agreement analysis between the My Jump 2^®^ app and force platform is depicted in Fig. 5. Our analysis did not reveal any systematic bias in the results obtained from either method. However, we did observe larger differences between the methods when compared to the observers.

## Discussion

The purpose of this study was to investigate the reliability and validity of the My Jump 2^®^ app in measuring jump height, flight time, and peak power on the sand surface among elite women beach volleyball players. The study provided evidence that the My Jump 2^**®**^ app is a valid and reliable tool for measuring jump height and flight time during a countermovement jump on a sand surface in elite women beach volleyball players.

Given the agreement between observers, the My Jump 2^**®**^ app showed to be highly reliable considering the between observers (*i.e*., inter-reliability). Like other study conducted on rigid surface (Balsalobre-Fernández, Glaister & Lockey, 2015), we found excellent agreement for flight time, jump height and peak power between observers. The mean differences for jump height, flight time, and peak power were only 0.18 cm, 1.59 ms, and 9.76 w, respectively, showing no significant differences for these variables. The Bland-Altman plot also revealed mean differences inter-observers for jump height similar to studies conducted on a rigid surface (Balsalobre-Fernández, Glaister & Lockey, 2015; Jimenez-Olmedo et al., 2022). However, it is noteworthy that a larger limit of agreement was found. Thus, the idea that the app reliability could be significantly impaired by surface softness was not confirmed.

Although we observed less agreement (ICC) between observers and force platform compared to CMJ jump height in rigid surface (0.99 *vs* 0.85) (Balsalobre-Fernández, Glaister & Lockey, 2015), there was a good agreement between the force platform and the My Jump 2^**®**^ app in measuring jump height, and flight time. However, we only found a moderate agreement for peak power (observers *vs* force platform). We did not find any significant differences between the values obtained for flight time, jump height, and peak power using My Jump 2^**®**^ app and the force platform.

The mean difference in jump height between the My Jump 2^**®**^ app and the force platform was only 0.32 cm, which was similar to the findings of another study (Gallardo-Fuentes et al., 2016). We also found small mean differences for flight time (5.87 ms) and peak power (−7.04 w). However, the coefficient of determination was very large for jump height and flight time, but only moderate for peak power.

The Bland-Altman plot indicated that the data obtained from the My Jump 2^**®**^ app and the force platform were close to the mean difference. The difference in jump height showed a limit of agreement between −4.10 and 4.74, indicating a high level of agreement between the two measurement tools (Martin Bland & Altman, 1986).

Some cautions should be considered while interpreting our findings because of some limitations. Firstly, the jump type can influence in the results. We only assessment the jumps with the hands on the hip. Second, we positioned the phone on 1.5 m from the force platform at floor level and sagittal plane, despite the app recommendations (record the feet from the front). Nevertheless, the height of the recording does not have any impact on the reliability and accuracy of the measurement (Koo & Li, 2016). However, we observed no significant differences between the observers using the My Jump 2^**®**^ app or between the My Jump 2^**®**^ app and the force platform. Based on these findings, we can suppose that the height of the recording and the sagittal plane did not significantly compromise the measurement. However, we cannot infer whether these adaptations reduce or increase the amount of variance explain by the My Jump 2^**®**^ app variables, especially for peak power. Finally, accounting for sand dissipation during take-off could potentially extend the flight time. This arises from athletes executing take-offs on a higher surface level compared to their landing. Future studies could consider recording the video in the sagittal plane to investigate if this would improve the agreement between the measurements and would include another jumps (drop jumps or jumps with free arms).

### Practical implications

In sports practiced on sand, the information provide by jumps on a rigid surface is limited, and using a sand box over a force platform is not feasible for most teams. Given its ease of use, portability, and data acquisition, this application can be used in daily practice by coaches to monitor jump capacity. Furthermore, high-level athletes and their teams who frequently travel for competitions can benefit from the convenience of the My Jump 2^®^ app, which enables easy assessment of the athlete’s jump performance in any location without the need for expensive equipment or logistical challenges.

## Conclusions

We concluded that My Jump 2^®^ app is a valid tool for measuring CMJ jump height and flight time on sand surfaces. However, more caution is needed when measuring peak power. Coaches can utilize the My Jump 2^®^ app to access jump variability, estimate neuromuscular recovery by analyzing the height of the jump, and monitor changes in jump performance pre- and post-competition.

## Supplemental Information

10.7717/peerj.17387/supp-1 Supplemental Information 1 Data.

## Additional Information and Declarations

### Data Availability

The following information was supplied regarding data availability: Raw data is available in the Supplemental File.
